# Editorial: Nutritional modulation of immune function in cancer

**DOI:** 10.3389/fnut.2023.1290026

**Published:** 2023-09-29

**Authors:** Fabiana Ourique, Maicon Roberto Kviecinski, Amanda Bagolin do Nascimento

**Affiliations:** ^1^Department of Biochemistry, Institute of Biological Sciences, Federal University of Juiz de Fora, Juiz de Fora, Minas Gerais, Brazil; ^2^Department of Biochemistry, Biological Sciences Center, Federal University of Santa Catarina, Florianópolis, Santa Catarina, Brazil; ^3^Department of Nutrition, Health Sciences Center, Federal University of Santa Catarina, Florianópolis, Santa Catarina, Brazil

**Keywords:** cancer, immune modulation, nutritional, dietary supplements, gut microbiota

According to the World Health Organization, one third of deaths caused by cancer are related to modifiable risk factors such as low consumption of fruit and vegetables, sedentary lifestyle, obesity, alcohol, and tobacco (1). In this context, the complex relationship between diet, nutrients and the immune system impacts on the prevention and therapy of diseases such as cancer. Dietary components are not only a source of nutrients for the maintenance of essential body functions, but they can also behave as antigens (2).

Nutrition and the immune system have a close relationship. Deviations from ideal body weight and alterations in essential micronutrients can highly influence the immune status. In the context of cancer, nutrition status can also influence the progression of diseases related to the immune system. Whilst chronic inflammation is a known risk factor for carcinogenesis, immune responses are needed to suppress cancer initiation and progress. Studies are therefore needed to explore in more depth how diet and nutrition products can help to balance immune responses either to prevent or treat cancer.

The complex cellular composition of the tumor microenvironment (TME) leads to a status of immune system evasion and chronic inflammation that promotes oncogenesis. Cancer cells, as well as tumor-associated macrophages and/or lymphocytes, from the activation of the transcription factors as NF-κB and STAT, produce pro-inflammatory cytokines such as interleukin (IL)-1, tumor necrosis factors (TNF) and IL-6, and C-reactive protein (3). In fact, once established, chronic inflammation drives the recruitment of fibroblasts contributing to the remodeling of TME and immunosuppression improving aggressiveness and invasiveness of tumor cells (4). Cancer-associated fibroblasts interact with immune components via secretion of growth factors, cytokines, chemokines, and other effector molecules, shaping an immunosuppressive TME and promoting tumor growth, invasion, angiogenesis, and metastasis (5). Tumor-associated macrophages lead to production of TGF-β that contribute to hypoxia and production of metalloproteinases which facilitating the metastatic profile of cancer cells (6, 7).

In the metabolically hostile TME, tumor-infiltrating immune cells adapt to various types of metabolic stress and can determine the immune surveillance, immunosuppression, and the adaptative chemoresistance (8). However, exogeneous agents such as diet or microbiota can modify the intra-tumor homeostasis promoting cancer-cell destruction from the modulation of the immune system to an antineoplastic inflammatory process (9, 10). In this regard, bioactive compounds derived from nutrition should be investigated to enhance the modulation of anti-tumor immune response.

The Research Topic *Nutritional modulation of immune function in cancer* aimed to promote and publish high quality articles on dietary supplements or biological compounds capable to correct nutritional status and affect immune function in cancer. We were contemplated with articles that investigated the effect of diet on the prognosis of cancer, studies that identified the influence of micronutrients extracted from different sources on circulating immune cells and molecules or other factors that can directly affect the tumor microenvironment, as well as article that report how nutritional factors may affect the gut microbiota by interfering with immune function in cancer.

Chen et al. showed that polymethoxyflavones extracted from *Citrus tangerine* cultivar “Dahongpao” inhibit the production of nitric oxide and biomarkers of chronic inflammation such as TNF-α and IL-6. Moreover, these bioactive compounds suppressed mRNA biomarkers of acute inflammation (*Cox-2* and *iNOS*). In addition, there was a significant decrease in the levels of pro-inflammatory cytokines (IL-4, IL-13, TNF-β, and IL-10). These effects caused an antiproliferative effect on human prostate cancer cell lines (PC-3 and DU145), and synergistically enhanced the cytotoxicity of mitoxantrone. These findings provide valuable insights into the anti-cancer activity of polymethoxyflavones.

Fang et al. compared the chemical properties and the anticancer activities of polysaccharides extracted from the sporoderm-removed spored of the medicinal mushroom *Ganoderma lucidum* (RSGLP) and polysaccharides extracted from the sporoderm-broken spores of *G. lucidum* (BSGLP). The results demonstrated that RSGLP was able to cause a decrease in cell viability and induce cell death by apoptosis in cancer cell lines of colon, liver, breast, and lung. The authors also noted that RSGLP was more effective in inhibiting xenograft tumor growth and inhibiting tumor-induced splenomegaly in nude mice, suggesting a better effect on immunomodulation of this bioactive compound. Finally, in nude mice, RSGLP inhibited serum inflammatory cytokines, and caused inhibitory effect *in vitro* on activation of macrophage and the expression of the inflammatory mediators, such as IL-1β, TNF-α, iNOS, and COX-2. The set of results suggests that polysaccharides extracted from the sporoderm-removed spores of *G. lucidum* can exert a promising immune-regulatory activity in cancer context.

Boucher et al. highlight that bioactive compounds from the diet play an important role in the regulation of the gut microbiota, and this microbiota plays a central role in anticancer immunomodulation, which can interfere with responsiveness to immunotherapy, as well as an ideal modulation for preventive and therapeutic purposes. In this regard, the authors showed that an inulin-enriched diet, a prebiotic know to promote immunostimulatory bacteria, triggers an enhanced in triggers an enhanced Th1-polarized CD4^+^ and CD8^+^ αβ T cell-mediated anti-tumor response and decreases the tumor growth in tumor-bearing mouse models. Furthermore, inulin causes an antitumor effect in a microbiota-dependent manner, since it induces the activation of intestinal ɤδ T cells and tumor infiltrators that are indispensable for the activation of αβ T cells that cause the control of tumor growth.

Hua et al. explored the relationship between lymphocyte-C-reactive protein ratio (LCR) and survival in patients with nasopharyngeal carcinoma. The authors introduced a novel LCR-based prognostic model, and it outperformed the conventional nasopharyngeal carcinoma staging system in terms of predictive power. However, the authors point out that further external verification remains necessary.

Finally, we truly hope that the reader will find in this Research Topic important references in the field of science involving nutrition, immune system, and cancer.

## Author contributions

FO: Writing—original draft, Writing—review and editing. MK: Writing—original draft, Writing—review and editing. AB: Writing—original draft, Writing—review and editing.
